# Comparison of different glycemic control indicators on incidence of acute kidney injury and long-term mortality in critically ill patients with atherosclerotic cardiovascular disease: A retrospective cohort study

**DOI:** 10.1371/journal.pone.0343234

**Published:** 2026-02-24

**Authors:** Dan Zhang, Lin Shi, Jia Wang

**Affiliations:** 1 Department of Nephrology, Affiliated Hospital of Xuzhou Medical University, Xuzhou, China; 2 Department of Gastroenterology, Xuzhou Central Hospital, Xuzhou Clinical School of Xuzhou Medical University, Xuzhou, Jiangsu, China; University of Diyala College of Medicine, IRAQ

## Abstract

**Background:**

Advanced glycemic metrics may better reflect glucose dysregulation in critically ill patients than conventional indicators, but their comparative predictive value for adverse outcomes in atherosclerotic cardiovascular disease (ASCVD) patients remains unclear.

**Methods:**

This retrospective study using the MIMIC-IV database assessed associations between stress hyperglycemia ratio (SHR), hemoglobin glycation index (HGI), and glycemic variability (GV) with acute kidney injury (AKI) and 365-day mortality in 2,820 ASCVD patients. Multivariable regression models, restricted cubic splines, and ROC curves evaluated predictive performance.

**Results:**

During ICU stay, 38.4% developed AKI, and 17.9% died within one year. GV showed the strongest association with both AKI (Q4 vs. Q1, OR=4.98, 95%CI:3.00–8.26, P < 0.001) and mortality (Q3 vs. Q1, HR = 1.77, 95%CI:1.19–2.63, P = 0.005). SHR was associated with increased AKI risk (Q4 vs. Q2, OR=1.47, 95%CI:1.12–2.19, P = 0.04) and mortality (Q4 vs. Q2, HR = 1.26, 95%CI:1.06–1.69, P = 0.01), while HGI showed inverse association with mortality (Q4 vs. Q1, HR = 0.71, 95%CI:0.53–0.93, P = 0.01). GV yielded the highest predictive accuracy for AKI (AUC = 0.69) and mortality (AUC = 0.62). Subgroup analyses confirmed robustness across demographics.

**Conclusions:**

Among critically ill ASCVD patients, GV outperformed SHR and HGI in predicting AKI and long-term mortality, underscoring its prognostic utility and supporting individualized glucose management in ICU settings.

## 1. Introduction

Atherosclerotic cardiovascular disease (ASCVD) remains the leading cause of global mortality, accounting for over 60% of cardiovascular deaths worldwide [1,2]. Critically ill ASCVD patients admitted to intensive care units (ICUs) frequently experience multi-organ dysfunction and carry substantial mortality risk [3]. Despite considerable advances in cardiovascular care and critical medicine over recent decades, mortality and complication rates among these patients remain alarmingly high [4]. Among the various adverse outcomes, acute kidney injury (AKI) has emerged as a particularly concerning complication that not only impacts short-term recovery but also significantly influences long-term mortality in this vulnerable population [5,6].

Stress-induced hyperglycemia is a common metabolic response in critically ill patients, occurring regardless of pre-existing diabetes status [7,8]. Both hyperglycemia and hypoglycemia have been consistently associated with adverse outcomes in ICU settings, including increased risk of AKI, infection, cardiovascular events, and mortality [9,10]. Large-scale evidence, including the NICE-SUGAR trial, has demonstrated that intensive glucose control increases mortality and hypoglycemia risk in ICU patients, highlighting the need for moderate glucose targets rather than tight control [11]. Recent guidance from the Society of Critical Care Medicine (SCCM 2024) further recommends moderate glycemic targets with frequent monitoring [12]. However, traditional glycemic metrics such as fasting glucose or glycated hemoglobin (HbA1c) may inadequately capture the complex dysregulation of glucose homeostasis during critical illness, particularly in ASCVD patients with altered metabolic profiles [13].

Recent attention has focused on more dynamic and individualized glucose metrics that may better reflect glycemic dysregulation in critical illness [13–16]. The Stress Hyperglycemia Ratio (SHR), calculated as the ratio of admission glucose to estimated average glucose derived from HbA1c, provides a more accurate representation of acute stress-induced hyperglycemia by accounting for chronic glycemic status [15]. The Hemoglobin Glycation Index (HGI) reflects inter-individual variation in glycation processes beyond ambient glucose exposure [13]. This metric has been validated in critical illness, where it distinguishes acute stress hyperglycemia from baseline glycemia [17]. Glycemic Variability (GV) captures fluctuations in glucose levels over time, which may trigger oxidative stress and endothelial dysfunction independent of mean glucose values [16]. These advanced indicators have demonstrated prognostic relevance in various cardiovascular conditions, including cardiac surgery, myocardial infarction, stroke, and sepsis [13–16,18,19].

Despite promising evidence, the comparative predictive value of these glycemic metrics for adverse outcomes in critically ill ASCVD patients remains unclear. Limited research has examined how these indices relate to incident AKI and long-term mortality in this high-risk population. Existing studies have either focused on single indicators or selected subgroups as a primary focus, creating a significant knowledge gap in this clinically important area [13–16,18,19].

Therefore, this study aimed to examine the associations between SHR, HGI, GV, and the incidence of AKI and long-term mortality among critically ill ASCVD patients. We sought to compare the predictive performance of these different glycemic indicators for these outcomes. Clarifying which glycemic metric best predicts adverse outcomes may improve risk stratification and guide individualized glucose management strategies in the ICU setting. These findings may support the development of tailored glucose targets and monitoring approaches for high-risk ASCVD patients, ultimately contributing to improved clinical outcomes in this vulnerable population.

## 2. Methods

### 2.1. Data source and study population

This retrospective cohort study utilized data from the MIMIC-IV database (version 3.1), a publicly accessible repository containing de-identified health records of 94,458 patients admitted to ICUs at Beth Israel Deaconess Medical Center between 2008 and 2022 [16,19]. The database includes detailed clinical information such as demographics, laboratory results, vital signs, medications, and outcomes, approved for research use by the Institutional Review Board (IRB) of the center. Access was granted to one co-author (Lin Shi, certification ID: 69047133) following completion of required training. Since the analyzed information was anonymized, patient informed consent was not necessary for this study.

We included adult patients (≥18 years) with a confirmed diagnosis of ASCVD, defined by International Classification of Diseases (ICD-9/10) codes, who were admitted to the ICU for the first time (The ICD codes were provided in S1 Table. Exclusion criteria comprised: (1) ICU readmissions during the same hospitalization (to avoid confounding by repeated exposures); (2) missing HbA1c or glucose data; (3) fewer than three glucose measurements during the ICU stay; and (4) liver cirrhosis or HIV. A flowchart detailing the inclusion and exclusion process will be provided in S1 Fig.

### 2.2. Data extraction

Data were extracted using PostgreSQL (version 16.0) and Structured Query Language (SQL) [20,21]. The following potential confounders were identified and extracted: (1) Demographic information, such as age, gender, and race; (2) Vital signs: heart rate, systolic blood pressure (SBP), diastolic blood pressure (DBP), SpO_2_, respiratory rate (RR); (3) Medications: use of ACEI/ARB, statins, β-blockers, insulin vasopressin, and continuous renal replacement therapy (CRRT); (4) Comorbidities: chronic kidney disease (CKD), chronic obstructive pulmonary disease (COPD), Hyperlipidemia, hypertension, diabetes mellitus, pneumonia and cancer; (5) Laboratory indices: white blood cells (WBC), platelets (PLT), hemoglobin (Hb), serum creatinine (Cr), blood urea nitrogen (BUN), serum potassium, serum sodium, hemoglobin A1c and glucose; and (6) severity of disease scores at the time of admission: sequential Organ Failure Estimate (SOFA) score, Simplified Acute Physiology Score II (SAPS II), and Charlson Comorbidity Index (CCI). Additionally, the length of ICU stay and the duration of hospitalization were considered. In the analysis model, variables with more than 10% missing values were excluded, and missing values were imputed using random forest interpolation.

### 2.3. Measurement and calculation of SHR, HGI and GV

In this study, the acute stress fasting glucose value was defined as the initial fasting blood glucose measurement obtained following the patient’s admission to the ICU [13]. The subsequent blood glucose values could be derived from various sources, including fingertip blood glucose tests, serum blood glucose assays, and whole blood glucose measurements. HGI quantifies the discrepancy between observed HbA1c and the HbA1c predicted from fasting plasma glucose (FPG), reflecting individual differences in hemoglobin glycation independent of mean glucose levels. Observed HbA1c was the first recorded value during ICU admission, measured in percentage units (%) [13]. SHR adjusts acute glucose levels for background glycemic control to assess the magnitude of stress-induced hyperglycemia. It was calculated using the formula: SHR = FPG/(28.7 × HbA1c − 46.7) [13,15]. GV measures fluctuations in blood glucose over the ICU stay, capturing instability in glycemic homeostasis. It was expressed as the coefficient of variation (CV), calculated by the mean of these measurements, and then multiplying by 100 (GV = (standard deviation/mean) × 100) [16,19]. GV was calculated using the coefficient of variation (CV) based on ≥3 glucose measurements within the first 24 hours after ICU admission.

### 2.4. Study endpoint

The primary clinical endpoint of this study was long-term all-cause mortality, specifically defined as death occurring within 365 days after ICU admission for acute severe cardiovascular disease. For all patients included in the analysis, the final follow-up was performed one year after their last hospital discharge. Mortality data were sourced from state or hospital death records. An additional clinical outcome assessed was the incidence of AKI. The diagnosis of AKI was determined according to the Kidney Disease [15,22]: Improving Global Outcomes (KDIGO) guidelines, which stipulate that at least one of the following criteria must be met: (1) an increase in serum creatinine (Scr) to ≥1.5 times the baseline level within the preceding 7 days; (2) an increase in Scr by ≥0.3 mg/dL (≥26.5 µmol/L) within a 48-hour period; or (3) urine output (UO) of less than 0.5 mL/kg/hour for 6 consecutive hours.

### 2.5. Statistical analysis

Patients were categorized into two groups based on long-term survival status. For the baseline data analysis, continuous variables that exhibited a normal distribution were presented as mean ± standard deviation. In contrast, continuous variables with a non-normal distribution were depicted using the median (interquartile range). Categorical variables were presented as frequency (percentage). Comparisons of continuous variables were conducted using either the two-tailed independent t-test or the Wilcoxon rank-sum test, contingent upon the normality of the data distribution. Normality was evaluated using the Shapiro-Wilk test. For categorical variables, analysis was performed using Pearson’s Chi-squared test or Fisher’s exact test, as appropriate. In our study, We categorized HGI, SHR, and GV into four groups based on their quartiles. (GV: Q1: < 14.83%, Q2: 14.83% to 21.25%, Q3: 21.25% to 31.75%, Q4: > 31.75%; SHR: Q1: < 0.89, Q2: 0.89 to 1.08, Q3: 1.08 to 1.34, Q4: > 1.34; HGI: Q1: < −0.78, Q2: −0.78 to −0.31, Q3: −0.31 to 0.35, Q4: > 0.35).

To investigate the relationship between HGI, SHR, and GV with the risk of AKI, we conducted univariate and multivariate logistic regression analyses, adjusting for relevant covariates. HGI, SHR, and GV were categorized based on quartiles. Model 1 was unadjusted, and Model 2 was adjusted for age, race, gender, diabetes, hypertension, CKD, SOFA score, SAPSII score, charlson score, hyperlipidemia. Model 3 included variables that showed statistical or clinical significance in the univariable analysis, after excluding those with multicollinearity using variance inflation factor and eigenvalue diagnostics. Model 3 adjusted for all variables in Model 2, plus SBP, DBP, RR, WBC, Hb, CRRT, statins, insulin, and β-blockers. Odds ratios (ORs) and 95% confidence intervals (CIs) were calculated to determine the impact of the three indictors on AKI. The dose-response relationship between HGI, SHR, and GV with the risk of AKI was further examined using restricted cubic splines (RCS), with adjustments for the same variables as in Model 3. In addition, receiver operating characteristic (ROC) curve analysis was performed to compare the predictive ability, sensitivity, and specificity of the three indices for assessing the risk of AKI.

To evaluate the association between HGI, SHR, and GV with long-term mortality across the entire cohort, both univariate and multivariable Cox proportional hazards regression models were utilized. The fully adjusted model incorporated the following covariates: age, gender, race, SBP, DBP, WBC, Hb, diabetes, hypertension, CKD, AKI, SOFA score, SAPSII score, charlson score, hyperlipidemia, CRRT, statins, insulin, and β-blockers. To explore the dose-relationship between the three indices (HGI, SHR, and GV) and mortality, RCS were also utilized. Furthermore, ROC curve analysis was conducted to evaluate and compare the predictive ability, sensitivity, and specificity of these three indices for assessing the risk of mortality. Statistical significance was assessed using a two-tailed p-value of 0.05.

## 3. Results

### 3.1. Baseline characteristics

A total of 2,820 critically ill patients with ASCVD admitted to the ICU were included in this study and the baseline characteristics of our study population were presented in Table 1. The mean age of the cohort was 69.78 ± 13.84 years, and 59.5% were male. The majority of participants were white (56.13%), followed by other races (34.89%) and black (8.97%). Non-survivors (n = 505) were older and exhibited higher ICU length of stay, faster heart and respiratory rates, and lower blood pressure compared to survivors (n = 2,315). Non-survivors also had significantly elevated glycemic indices, including glucose levels, GV, and SHR, and reduced HGI values. Additionally, they showed higher white blood cell counts, creatinine, and urea nitrogen, alongside lower hemoglobin concentrations (P < 0.001). Comorbidities such as diabetes, pneumonia, chronic kidney disease, and AKI were more frequent among non-survivors. Disease severity scores (SOFA, SAPSII, Charlson) were consistently higher in non-survivors (all P < 0.001). Non-survivors had a higher prevalence of diabetes, COPD, pneumonia, CKD, and AKI (all P < 0.05). They more frequently required continuous renal replacement therapy (14.46% vs. 2.68%, P < 0.001) and vasoactive agents (70.89% vs. 42.33%, P < 0.001), but less often received beta-blockers (47.8% vs. 68.8%, P < 0.001) and insulin (65.4% vs. 70.9%, P = 0.01).

**Table 1:** Baseline characteristics of study population.

Variables	Total (n = 2820)	Survivors (n = 2315)	Non-survivors (n = 505)	P
Age, year	69.78 ± 13.84	69.30 ± 14.03	71.98 ± 12.69	<.001
Male, n(%)	1678 (59.50)	1380 (59.61)	298 (59.01)	0.803
Race, n(%)				<0.001
White	1583 (56.13)	1347 (58.19)	236 (46.73)	
Black	253 (8.97)	206 (8.90)	47 (9.31)	
Other	984 (34.89)	762 (32.92)	222 (43.96)	
Length of hospitalization, days	10.17 (6.15, 17.78)	10.17 (6.23, 17.49)	10.17 (5.59, 18.86)	0.265
Length of ICU, days	3.81 (2.05, 7.00)	3.62 (2.02, 6.61)	5.06 (2.56, 9.70)	<.001
Vital signs				
Heart rate, bpm	83.00 (72.00, 98.25)	82.00 (71.00, 97.00)	88.00 (74.00, 103.00)	<.001
SBP, mmHg	130.00 (112.00, 149.00)	131.00 (113.00, 150.00)	123.00 (107.25, 142.75)	<.001
DBP, mmHg	73.00 (62.00, 86.00)	74.00 (63.00, 87.00)	71.00 (59.00, 83.00)	<.001
Respiratory rate, breaths/minute	19.00 (16.00, 23.00)	19.00 (16.00, 22.00)	20.00 (16.00, 24.00)	<.001
Spo2, %	98.00 (95.00, 100.00)	98.00 (95.00, 100.00)	98.00 (95.00, 100.00)	0.392
Lab				
Hemoglobin A1c	5.90 (5.40, 6.80)	5.90 (5.40, 6.80)	6.00 (5.50, 6.90)	0.005
Glucose, mg/dL	135.00 (108.00, 181.00)	132.00 (106.00, 177.00)	152.00 (120.00, 210.00)	<.001
Hemoglobin, g/dL	11.80 (10.00, 13.40)	12.00 (10.30, 13.45)	11.10 (9.30, 12.80)	<.001
Platelet Count, K/uL	207.00 (159.75, 265.00)	208.00 (162.00, 263.00)	203.00 (147.00, 272.00)	0.282
White Blood Cells, K/uL	10.80 (8.10, 14.30)	10.50 (8.00, 13.70)	12.40 (8.90, 16.92)	<.001
Potassium, mEq/L	4.10 (3.80, 4.50)	4.10 (3.77, 4.50)	4.20 (3.80, 4.80)	<.001
Sodium, mEq/L	139.00 (136.00, 141.00)	139.00 (136.00, 141.00)	138.00 (135.00, 141.00)	0.045
Creatinine, mg/dl	1.00 (0.80, 1.40)	1.00 (0.80, 1.40)	1.20 (0.90, 2.00)	<.001
Urea Nitrogen, (mg/dL)	19.00 (14.00, 30.00)	18.00 (13.00, 28.00)	26.00 (18.00, 45.00)	<.001
GV(%)	20.21 (14.16, 30.64)	19.15 (13.61, 29.29)	24.88 (17.13, 34.84)	<.001
SHR	1.07 (0.89, 1.33)	1.05 (0.88, 1.30)	1.19 (0.93, 1.50)	<.001
HGI	0.04 ± 1.55	0.06 ± 1.56	−0.06 ± 1.49	0.049
Score system				
SOFA	4.00 (2.00, 6.00)	3.00 (2.00, 5.00)	5.00 (3.00, 8.00)	<.001
SAPSII	35.00 (27.00, 43.00)	33.00 (26.00, 41.00)	41.00 (34.00, 50.00)	<.001
Charlson	6.00 (5.00, 8.00)	6.00 (4.00, 8.00)	7.00 (6.00, 9.00)	<.001
Comorbidities, n(%)				
Hypertension	1250 (44.33)	1061 (45.83)	189 (37.43)	<.001
Diabetes	1108 (39.29)	887 (38.32)	221 (43.76)	0.023
Hyperlipidemia	1466 (51.99)	1246 (53.82)	220 (43.56)	<.001
COPD	405 (14.36)	318 (13.74)	87 (17.23)	0.043
Pneumonia	827 (29.33)	605 (26.13)	222 (43.96)	<.001
CKD	657 (23.30)	500 (21.60)	157 (31.09)	<.001
Cancer	348 (12.34)	275 (11.88)	73 (14.46)	0.111
AKI	1083 (38.40)	780 (33.69)	303 (60.00)	<.001
Medications, n(%)				
ACEI/ ARB	1664 (59.23)	1364 (58.9)	300 (59.4)	0.76
B-blockers	1838 (65.21)	1597 (68.8)	241 (47.8)	<0.001
Insulin	1974 (70.11)	1642 (70.9)	332 (65.4)	0.01
Statin	2538 (90.46)	2082 (89.9)	456 (90.3)	0.13
CRRT	135 (4.79)	62 (2.68)	73 (14.46)	<0.001
Vasoactive agent	1338 (47.45)	980 (42.33)	358 (70.89)	<0.001

Continuous numerical variables are expressed as medians (interquartile spacing) and categorical variables are expressed as numbers (percentages).

SBP, systolic blood pressure, DBP, diastolic blood pressure, SpO2, oxyhemoglobin saturation, SOFA, sequential organ failure assessment; SAPSII, simplified acute physiology score II; HGI, hemoglobin glycation index; SHR, stress hyperglycemia ratio; GV, glycemic variability, COPD, chronic kidney disease; AKI, acute kidney injury; COPD: chronic obstructive pulmonary disease; ACEI, angiotensin-converting enzyme inhibitions, ARB, angiotensin receptor blockers; CRRT: continuous renal replacement therapy.

When stratified by the presence of AKI, significant differences were observed in glycemic indices. Among the 2,820 critically ill ASCVD patients, 1,083 (38.4%) developed AKI during their ICU stay. Patients with AKI exhibited significantly higher glycemic parameters compared to those without AKI, including elevated GV (24.88 vs. 19.15, P < 0.001) and SHR (1.19 vs. 1.05, P < 0.001). HGI was also higher in the AKI group (0.10 ± 1.74 vs. −0.00 ± 1.39, P = 0.003). Additionally, patients with AKI had significantly higher mortality rates (27.98% vs. 11.63%, P < 0.001). Other clinical details can be found in S2 Table.

**Fig 1:**
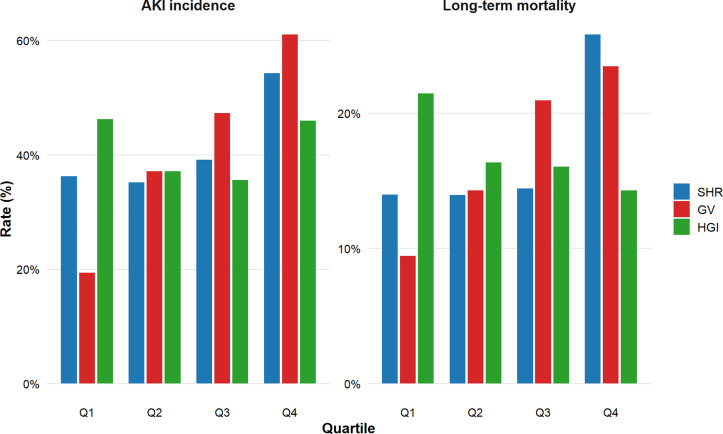
The incidence of AKI and long-term mortality bar graphs based on quartiles of SHR, GV and HGI stratification. Abbreviations: SHR stress hyperglycemia ratio, GV glycemic variability. HGI, hemoglobin glycation index.

To further explore the association between different glycemic indices and clinical outcomes, we stratified GV, SHR, and HGI into quartiles and plotted the corresponding rates of AKI incidence and long-term mortality. As shown in Fig 1, GV demonstrated the strongest dose–response relationship, with the incidence of AKI rising sharply from Q1 to Q4, peaking at over 60% in the highest quartile. A similar pattern was observed for long-term mortality. SHR exhibited a milder, more gradual increase in both AKI and mortality risk across quartiles. In contrast, HGI showed an inconsistent pattern without a clear gradient, suggesting a less robust association with adverse outcomes.

### 3.2. Association between HGI, SHR and GV with risk of AKI

In our study, a total of 1083 (52.07%) patients with ASCVD experienced AKI (S2 Table). As shown in Table 2, we evaluated the associations of HGI, SHR, and GV with the incidence of AKI using multivariate logistic regression models across quartile groups. In the fully adjusted model (Model 3), SHR demonstrated a clear dose–response relationship with AKI risk. Compared with the reference group (Q2), patients in the highest quartile of SHR (Q4) had a significantly increased risk of developing AKI (OR = 1.47, 95% CI: 1.12–2.19, P = 0.04). As shown in Table 2, in the fully adjusted model, HGI indicated a non-linear relationship but with no statistically significant association with the incidence of AKI (P > 0.05). GV was strongly and positively associated with AKI occurrence. Compared to the Q1 group, patients in Q3 and Q4 had significantly elevated odds of AKI (Q3: OR = 3.52, 95% CI: 2.12–5.83, P = 0.001; Q4: OR = 4.98, 95% CI: 3.00–8.26, P < 0.001). These findings suggest that among the three glycemic indicators, GV exhibited the most consistent and robust association with AKI risk across quartiles.

**Table 2:** Multivariate logistic regression analysis of incidence of AKI in the SHR groups, HGI groups, and GV groups.

Exposure	Model 1; OR (95% CI, P)	Model 1; OR (95% CI, P)	Model 1; OR (95% CI, P)
SHR group			
Q1	0.77 (0.61, 0.96), P = 0.02	0.73(0.57, 0.93), P = 0.02	0.84 (0.55, 1.28), P = 0.43
Q2	Ref	Ref	Ref
Q3	1.06 (0.79, 1.243), P = 0.9	0.91(0.72, 1.16), P = 0.45	1.03 (0.68, 1.55), P = 0.89
Q4	1.91 (1.52,2.36), P < 0.001	1.31(1.03, 1.65), P = 0.03	**1.47(1.12, 2.19), P = 0.04**
HGI group			
Q1	0.572 (0.45, 0.71), P < 0.001	0.73(0.55, 0.93), P = 0.01	1.12(0.76, 1.63), P = 0.56
Q2	Ref	Ref	Ref
Q3	0.61 (0.49, 0.76), P < 0.001	0.81(0.631,1.02), P = 0.07	0.92(0.62,1.36), P = 0.68
Q4	1.06 (0.85, 1.32), P = 0.57	1.421 (1.12, 1.79), P = 0.003	1.26 (0.84, 1.86), P = 0.26
GV group			
Q1	Ref	Ref	Ref
Q2	2.31(1.78, 2.97), P < 0.001	1.98(1.52, 2.59), P < 0.001	**2.74 (1.65, 4.57), P < 0.001**
Q3	3.56(2.77, 4.57), P < 0.001	2.87(2.21, 3.72), P < 0.001	**3.52 (2.12, 5.83), P = 0.001**
Q4	6.75(5.26,8.67), P < 0.001	4.72(3.62, 6.21), P < 0.001	**4.98 (3.00, 8.26), P = 0.001**

Bold represents statistically significant correlation with the outcome and p-value less than 0.05 in the fully adjusted model.

Model 1: no covariates were adjusted

Model 2: adjusted for age, gender, race, diabetes, hypertension, CKD, SOFA score, SAPSII score, charlson score, hyperlipidemia.

Model 3: adjusted for Model 2 plus SBP, DBP, RR, WBC, Hb, potassium, sodium, urea nitrogen, statins, insulin, and β-blockers.

**Fig 2:**
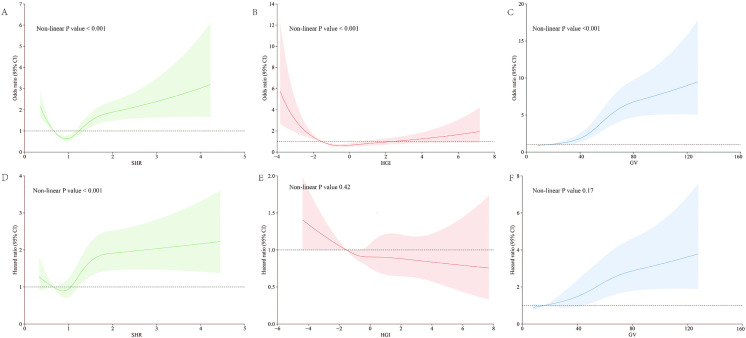
Association of SHR, HGI and GV with incidence of AKI and long-term mortality among critically ill patients with atherosclerotic cardiovascular disease. A–C: Restricted Cubic Spline Curve for incidence of AKI among critically ill patients with atherosclerotic cardiovascular disease. Fully adjusted ORs are indicated by blue lines for SHR cohort, red lines for HGI cohort, and by green lines for SHR cohort, shaded areas indicate 95% CIs. D–F: Restricted Cubic Spline Curve for the long-term mortality rate of patients. Fully adjusted HRs are indicated by blue lines for SHR cohort, red lines for HGI cohort, and by green lines for SHR cohort, shaded areas indicate 95% CIs. Abbreviations: CI confidence interval, HR hazard ratio, HGI hemoglobin glycation index, SHR stress hyperglycemia ratio, GV glycemic variability.

We employed RCS analysis to examine the associations between HGI, SHR, and GV with long-term mortality, based on Model 3. As depicted in Fig 2, both HGI and SHR demonstrated a non-linear relationship with AKI in patients with ASCVD (P < 0.001; Fig 2A and 2B), which aligns with the results from logistic regression analysis. Conversely, GV showed a linear association with AKI, as indicated by a non-linearity P value greater than 0.05 (Fig 2C).

### 3.3. Association between HGI, SHR and GV with risk of mortality

During the 365-day follow-up, 505 patients (17.9%) died (Table 1). As shown in Table 3, the associations between quartiles of HGI, SHR, and GV and the risk of 365-day all-cause mortality were assessed using multivariate Cox proportional hazards models. For SHR, using Q2 as the reference group, only the highest quartile (Q4) was significantly associated with increased mortality risk (HR = 1.26, 95% CI: 1.06–1.69, P = 0.01), whereas the intermediate quartiles (Q2 and Q3) showed no significant associations, suggesting a modest but non-linear relationship between SHR and long-term mortality. In contrast, the relationship between HGI and mortality appeared inverse. Compared to the lowest quartile (Q1), the highest quartile of HGI (Q4: HGI) was significantly associated with a lower risk of death (HR = 0.71, 95% CI: 0.53–0.93, P = 0.01). Although the second and third quartiles (Q2 and Q3) also showed a trend toward reduced risk, the differences were not statistically significant. GV was positively associated with long-term mortality in a dose-dependent manner. Compared to the reference group (Q1), both Q3 and Q4 were significantly associated with higher mortality risks (Q3: HR = 1.77, 95% CI: 1.19–2.63, P = 0.005; Q4: HR = 1.55, 95% CI: 1.04–2.31, P = 0.03), whereas Q2 did not reach statistical significance. Among the three glycemic markers, GV demonstrated the strongest and most consistent predictive value for adverse long-term outcomes.

**Table 3:** Multivariate Cox analysis of long-term mortality in the SHR groups, HGI groups, and GV groups.

Exposure	Model 1; HR (95% CI, P)	Model 1; HR (95% CI, P)	Model 1; HR (95% CI, P)
SHR group			
Q2	Ref	Ref	Ref
Q1	1.02(0.77,1.34), P = 0.92	1.02(0.78,1.35), P = 0.85	1.04(0.76, 1.43), P = 0.81
Q3	1.10(0.84,1.45), P = 0.47	1.14 (0.87,1.51), P = 0.33	0.87(0.62, 1.19), P = 0.38
Q4	1.78(1.38,2.28), P = 0.001	1.81(1.41,2.32), P = 0.001	**1.26(1.06, 1.69), P = 0.01**
HGI group			
Q1	Ref	Ref	Ref
Q2	0.83(0.64, 1.05), P = 0.11	0.82(0.64, 1.05), P = 0.11	0.81 (0.61, 1.05), P = 0.12
Q3	0.78(0.61, 0.99), P = 0.046	0.72(0.56, 0.92), P = 0.01	0.88 (0.67, 1.17), P = 0.39
Q4	0.68(0.55, 0.92), P = 0.008	0.69(0.54, 0.88), P = 0.004	**0.71 (0.53, 0.93), P = 0.01**
GV group			
Q1	Ref	Ref	Ref
Q2	1.53(1.18, 1.99), P = 0.001	1.53(1.17, 1.99), P = 0.002	1.32(0.87, 1.99), P = 0.19
Q3	2.32(1.81, 2.97), P = 0.000	2.32 (1.81, 2.96), P = 0.001	**1.77(1.19, 2.63), P = 0.005**
Q4	2.61(2.05, 3.32), P = 0.000	2.64 (2.07, 3.36), P = 0.001	**1.55(1.04, 2.31), P = 0.03**

Bold represents statistically significant correlation with the outcome and p-value less than 0.05 in the fully adjusted model.

Model 1: no covariates were adjusted,

Model 2: adjusted for age, gender, race, diabetes, hypertension, CKD, SOFA score, SAPSII score, charlson score, hyperlipidemia.

Model 3: adjusted for Model 2 plus SBP, DBP, RR, WBC, Hb, potassium, sodium, urea nitrogen, CRRT, AKI, statins, insulin, and β-blockers.

We conducted Survival-RCS analysis to evaluate the associations between HGI, SHR, and GV with long-term mortality, based on Model 3. As illustrated in Fig 2, SHR exhibited a non-linear relationship with long-term mortality risk in patients with ASCVD (P < 0.001; see Fig 2D). Therefore, the second quartile (Q2) was used as the reference category. Additionally, HGI also showed a near linear relationship as the risk of mortality decreased continuously across the range of HGI values (Fig 2E). In contrast, GV displayed a linear association with long-term mortality, as evidenced by a non-linearity P value greater than 0.05 (Fig 2F).

### 3.4. Subgroup analysis and ROC curve analysis of HGI, SHR and GV

Subgroup analyses were conducted to examine associations of SHR, HGI, and GV with AKI incidence and long-term mortality in critically ill patients with atherosclerotic cardiovascular disease, stratified by age, sex, diabetes, hypertension, and CKD (S3–S5 Tables).

As shown in S3 Table, higher SHR (Q4 vs. Q2) was associated with increased AKI risk, particularly in patients without diabetes (OR 1.96, 95% CI 1.30–2.96, P = 0.001) or hypertension (OR 1.87, 95% CI 1.40–2.51, P < 0.0001). No significant interactions were observed. S4 Table indicates that lower HGI (Q1 vs. Q2) reduced AKI risk in patients aged <60 years (OR 0.56, 95% CI 0.34–0.93, P = 0.03). Long-term mortality associations were weaker, with no significant interactions. For S5 Table, higher GV (Q4 vs. Q1) was strongly associated with AKI across subgroups, especially in females (OR 6.12, 95% CI 3.79–9.89, P < 0.0001) and patients without diabetes (OR 4.98, 95% CI 3.50–7.09, P < 0.0001). Higher GV also increased long-term mortality risk, particularly in patients without diabetes (HR 2.36, 95% CI 1.63–3.41, P < 0.0001). No significant interactions were found. Overall, GV showed the strongest associations with AKI and mortality, while SHR and HGI were particularly predictive of AKI in patients without diabetes or hypertension.

**Fig 3:**
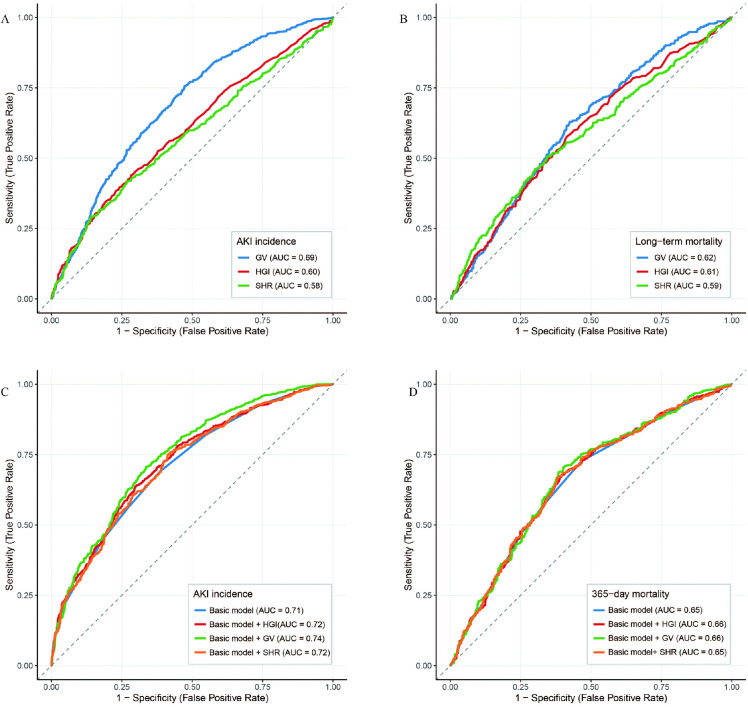
The ROC curves of the SHR, HGI, and GV to predict incidence of AKI and long-term mortality. A: SHR versus HGI versus GV to predict AKI. B: SHR versus HGI versus GV to predict long-term mortality. C: Basic risk model versus +SHR, + HGI, + GV to predict AKI. D: Basic risk model versus +SHR, + HGI, + GV to predict long-term mortality.

ROC curves evaluating the predictive performance of HGI, SHR, and GV for AKI and long-term mortality in critically ill patients with atherosclerotic cardiovascular disease are shown in Fig 3A and 3B and S6 Table. For AKI, GV exhibited the highest area under the curve (AUC) of 0.69 (95% CI 0.66–0.71), outperforming HGI (AUC 0.60, 95% CI 0.58–0.62) and SHR (AUC 0.58, 95% CI 0.56–0.60; all P < 0.001). For long-term mortality, GV again showed superior predictive ability (AUC 0.62, 95% CI 0.58–0.63) compared to HGI (AUC 0.59, 95% CI 0.57–0.61) and SHR (AUC 0.59, 95% CI 0.56–0.61; all P < 0.001). Optimal cutoffs were 18.66 for GV predicting AKI (sensitivity 0.76, specificity 0.53) and 21.53 for long-term mortality (sensitivity 0.65, specificity 0.54).

Finally, we assessed whether the inclusion of glycemic control indicators would further enhance the predictive ability of the basic model (Model 3). The AUC values and p-values for comparison are presented in Fig 3C and 3D and Table 4. The results demonstrated that the addition of these three glycemic control indicators provided a moderate increase in predictive ability to the basic risk model in patients with ASCVD.

**Table 4:** Improvement in discrimination for mortality after adding glucose indices.

Models	AUC (95% CI)	P value
AKI incidence		
Basic model	0.710 (0.694–0.726)	
+HGI	0.723 (0.707–0.739)	<0.001
+GV	0.743 (0.728–0.758)	<0.001
+SHR	0.715 (0.699–0.731)	<0.001
Long-term mortality		
Basic model	0.654 (0.632–0.677)	
+HGI	0.659 (0.637–0.681)	0.07
+GV	0.663 (0.641–0.686)	0.02
+SHR	0.659 (0.637–0.681)	0.06

The basic model for predicting AKI included age, gender, race, diabetes, hypertension, CKD, SOFA score, SAPSII score, charlson score, hyperlipidemia, SBP, DBP, RR, WBC, Hb, potassium, sodium, urea nitrogen, statins, insulin, and β-blockers (Model 3).

The basic model for predicting mortality included age, gender, race, diabetes, hypertension, CKD, SOFA score, SAPSII score, charlson score, hyperlipidemia, SBP, DBP, RR, WBC, Hb, potassium, sodium, urea nitrogen, CRRT, AKI, statins, insulin, and β-blockers (Model 3).

## 4. Discussion

Our study aimed to compare the predictive value of different glycemic control indicators—SHR, HGI, and GV—for acute kidney injury and long-term mortality in critically ill patients with ASCVD. Our findings demonstrate that GV exhibited the strongest and most consistent association with both AKI and 365-day mortality compared to other glycemic indicators. Specifically, patients in the highest quartile of GV had nearly five times the risk of developing AKI compared to the lowest quartile (OR = 4.98, P < 0.001) and a significantly higher risk of mortality (HR = 1.55, P = 0.03). SHR showed a significant association with increased AKI risk (Q4 vs. Q2, OR = 1.47, P = 0.04) and mortality (Q4 vs. Q2, HR = 1.26, P = 0.01), while HGI unexpectedly demonstrated an inverse association with mortality (Q4 vs. Q1, HR = 0.71, P = 0.01). Importantly, when these glycemic indicators were sequentially added to our baseline risk model (Model 3), the addition of GV provided the most substantial improvement in predictive performance for both AKI and mortality outcomes. GV consistently provided the highest predictive accuracy for both outcomes (AUC = 0.69 for AKI, AUC = 0.62 for mortality), and these findings remained robust across various subgroup analyses by age, sex, and diabetes status.

Glycemic control holds particular significance in critically ill ASCVD patients due to the complex interplay between glucose metabolism and cardiovascular pathophysiology [7–10]. Acute illness triggers a stress response characterized by insulin resistance and hyperglycemia, which can exacerbate oxidative stress and inflammation in an already compromised cardiovascular system [23–28]. For ASCVD patients, whose vasculature is inherently vulnerable due to atherosclerotic changes, dysregulated glucose metabolism can further impair endothelial function, increase platelet aggregation, and promote thrombosis [25,26]. Additionally, fluctuations in glucose levels can destabilize atherosclerotic plaques and trigger acute coronary syndromes. Proper glycemic management in these patients is not merely about controlling absolute glucose levels but maintaining metabolic stability that minimizes cellular stress and organ damage [10,23,24]. Our findings suggest that beyond traditional glycemic targets, the pattern and stability of glucose levels may be crucial determinants of outcomes in this high-risk population. By identifying GV as the most powerful predictor among glycemic indicators, our study highlights the potential importance of glucose stabilization strategies in improving both short-term organ protection (reducing AKI) and long-term survival in critically ill ASCVD patients.

Glycemic variability emerged as the most powerful predictor of adverse outcomes in our study, showing a strong dose-response relationship with both AKI occurrence and long-term mortality. This finding aligns with previous research suggesting that glucose fluctuations may be more detrimental than sustained hyperglycemia in critical illness [16,19,29,30]. The pathophysiological mechanisms underlying this association likely involve amplified oxidative stress, endothelial dysfunction, and inflammatory responses triggered by rapid glucose fluctuations [31,32]. Compared to sustained hyperglycemia, oscillatory glucose levels exert greater harmful effects on endothelial cells by promoting monocyte adhesion to endothelial cells, accelerating endothelial apoptosis, and enhancing the production of reactive oxygen species [31]. In ASCVD patients, whose vasculature is already compromised, these rapid glucose oscillations may exacerbate microcirculatory dysfunction and cellular injury in the kidneys, contributing to AKI development [32,33]. Recent studies in various populations have similarly identified GV as an independent risk factor for cardiovascular events and mortality [34,35]. For instance, research from heart failure registries has shown that increased GV correlates with higher all-cause mortality during follow-up, particularly among patients without diabetes [34]. The linear relationship between GV and outcomes in our restricted cubic spline analysis further emphasizes the clinical significance of even modest increases in glucose fluctuations, suggesting that GV may be a more sensitive indicator of dysregulated glucose metabolism than traditional metrics in critically ill populations.

SHR demonstrated a significant association with both AKI and mortality, particularly in the highest quartile (Q4), suggesting a threshold effect rather than a continuous relationship. As a metric that accounts for baseline glycemic status, SHR captures the relative hyperglycemia induced by acute physiological stress [15]. The observed non-linear relationship between SHR and outcomes, as confirmed by our RCS analysis, suggests that mild to moderate stress hyperglycemia may represent an appropriate adaptive response, while excessive hyperglycemia relative to baseline reflects dysregulated glucose homeostasis and predicts poorer outcomes. The significance of stress-related states is particularly pronounced in patients with severe cardiovascular diseases, and SHR, adjusted for baseline glucose levels, is considered a more effective biomarker for stress hyperglycemia than absolute blood glucose. This aligns with studies by Roberts et al., who reported that SHR outperformed absolute glucose levels in predicting adverse outcomes in critically ill patients [17]. Previous investigations have identified SHR as a strong predictor of adverse cardiovascular outcomes and increased hospital mortality, particularly in heart attack and heart failure cases [8,15,32]. The stronger association of SHR with AKI in non-diabetic patients observed in our subgroup analysis further suggests that acute glycemic derangements may be particularly harmful in those without preexisting glucose dysregulation, potentially because the internal environment of non-diabetic patients is less adapted to the inflammatory and oxidative stress responses caused by sudden increases in serum glucose.

Interestingly, our analysis revealed an inverse relationship between HGI and long-term mortality, with the highest HGI quartile showing significantly lower mortality risk compared to the lowest quartile. The relationship between HGI and AKI demonstrated a U-shaped curve, suggesting that both lower and higher HGI values might increase the risk of AKI. However, after multivariate analysis, the association between HGI and AKI lacked statistical significance, indicating that the relationship between HGI and both AKI and prognosis is not as strong as that of SHR and GV. Over the past twenty years, numerous studies have examined the connection between HGI and various cardiovascular disease risks, with some reporting a “U-shaped” association where both very low and high HGI values correlated with increased cardiovascular events [36–38]. Similarly, another study demonstrated that both low and high HGI were linked to poor long-term outcomes in critically ill patients with coronary artery disease [38]. In our study, we discovered for the first time that among ASCVD patients, HGI showed no statistically significant relationship with AKI incidence; however, higher HGI values might reduce long-term mortality in ASCVD patients, which is consistent with previous research findings [13,18]. The lack of significant association between HGI and AKI risk in our study further suggests this metric may be less relevant for acute complications during critical illness, potentially limiting its usefulness in guiding short-term management decisions.

Our findings have several important clinical implications for the management of critically ill ASCVD patients. First, they suggest that conventional glycemic monitoring focusing solely on mean glucose levels may be insufficient to identify patients at highest risk for adverse outcomes. Incorporating GV assessment into routine ICU monitoring could enhance risk stratification and guide more targeted interventions. Second, our results support the potential benefit of glucose stabilization strategies over strict glycemic control protocols that may inadvertently increase hypoglycemic events and glucose fluctuations [7,10]. Implementation of continuous glucose monitoring systems in ICU settings could facilitate real-time tracking of glucose fluctuations and enable prompt interventions to minimize variability. Finally, our finding that glycemic metrics provide additive predictive value to established risk models suggests that integration of these parameters into clinical decision support tools could improve prognostication and resource allocation for critically ill ASCVD patients.

Our study has several strengths that enhance the validity and clinical relevance of our findings. First, this is the first study to explore the association of HGI, SHR, and GV on the incidence of AKI and 365-day mortality of ASCVD patients based on a large sample of databases with follow-up outcome. Second, we comprehensively evaluated multiple glycemic indicators simultaneously, allowing direct comparison of their predictive performance. Third, our statistical methodology was robust, employing multivariable regression models with extensive adjustment for confounders, RCS analysis to explore non-linear relationships, and ROC curve analysis to assess predictive accuracy. Additionally, we examined both short-term (AKI) and long-term (365-day mortality) outcomes, providing a comprehensive assessment of the prognostic value of these glycemic metrics across different time horizons. Our focus on ASCVD patients specifically addresses an important high-risk subgroup within the critically ill population.

Despite these strengths, several limitations warrant consideration. First, the retrospective nature of our study precludes establishment of causal relationships between glycemic indicators and outcomes. Second, we lacked information on pre-admission glycemic control and diabetes management, which could confound the observed associations. Third, selection bias is possible because patients without available HbA1c or sufficient glucose measurements were excluded. Fourth, our assessment of GV relied on point-of-care measurements rather than continuous glucose monitoring, potentially underestimating the true extent of glucose fluctuations. Additionally, residual confounding from unmeasured factors such as steroid exposure, nutritional strategies, or fluid balance cannot be excluded. Fifth, our AKI definition was based on the KDIGO criteria, but incomplete urine output data may have led to misclassification. Sixth, this study was conducted using the single-center MIMIC-IV database, which may limit generalizability to other ICU settings or health systems. Future prospective studies should address these limitations by implementing continuous glucose monitoring, standardizing measurement protocols, and collecting comprehensive pre-admission data. Additionally, this study was limited to patients with ASCVD who were admitted to the ICU and did not include those with ASCVD who bypassed the ICU. Future research should examine the relationship between blood glucose control indicators and the prognosis of ASCVD patients who did not require ICU admission. Moreover, it would be valuable to compare these findings with those of patients who were admitted to the ICU.

## 5. Conclusion

In conclusion, GV showed stronger associations with AKI and long-term mortality than SHR and HGI in critically ill ASCVD patients. These findings highlight the prognostic value of glucose fluctuations, although their discrimination is modest and should be interpreted within guideline-recommended, moderate glycemic targets rather than as intervention goals.

## Supporting information

S1 Table Disease codes included in the study. (DOCX)

S2 Table Baseline characteristics of study population according to the incidence of AKI. (DOCX)

S3 Table Association between SHR with AKI and long-term mortality in different subgroups. (DOCX)

S4 Table Association between HGI with AKI and long-term mortality in different subgroups. (DOCX)

S5 Table Association between GV with AKI and long-term mortality in different subgroups. (DOCX)

S6 Table Discrimination of each predictive model for outcomes. (DOCX)

S1 Fig The flow-chart of our study. (TIF)
