# Supplementary material for: Comparison of different glycemic control indicators on incidence of acute kidney injury and long-term mortality in critically ill patients with atherosclerotic cardiovascular disease: A retrospective cohort study
Source: PLoS One. 2026 Feb 24;21(2):e0343234. doi: 10.1371/journal.pone.0343234 (PMC12931771; doi:10.1371/journal.pone.0343234)
Supplement: S1 Table — (DOCX) [file pone.0343234.s001.docx]

Table S1 Disease codes included in the study

| Ischemic stroke | ICD9 | 433.00,433.01,433.10,433.11,433.20,433.21,433.30,433.31,433.80,433.81,433.90,433.91,434.00,434.01,434.10,434.11,434.90,434.91 |
| --- | --- | --- |
|  | ICD10 | I63.41, I63.42, I63.43, I63.44, I63.5, I63.51, I63.519, I63.52, I63.53, I63.54, I65, I66, I63.40, I63.411, I63.412, I63.413, I63.419, I63.421, I63.422, I63.423, I63.429, I63.431, I63.432, I63.433, I63.439, I63.441, I63.442, I63.443, I63.449, I63.9, I63.50, I63.511, I63.512, I63.513, I63.521, I63.522, I63.523, I63.529, I63.531, I63.532, I63.533, I63.539, I63.541, I63.542, I63.543, I63.549, I63.9, I63.6, I63.8, I63.81, I63.89, I63.9 |
| Heart disease | ICD10 | I25.5, I20.0, I20.1, I20.8, I20.9, I21.0, I21.01, I21.02, I21.09, I21.11, I21.19, I21.21, I21.29, I21.3, I21.4, I21.9, I21.A1, I21.A9, I22.0, I22.1, I22.2, I22.8, I22.9, I23.0, I23.1, I23.2, I23.3, I23.4, I23.5, I23.6, I23.7, I23.8, I24.0, I24.1, I24.8, I24.9, I25.10, I25.110, I25.111, I25.118, I25.119, I25.810, I25.82, I25.83, I25.84 |
