# Supplementary material for: Comparison of different glycemic control indicators on incidence of acute kidney injury and long-term mortality in critically ill patients with atherosclerotic cardiovascular disease: A retrospective cohort study
Source: PLoS One. 2026 Feb 24;21(2):e0343234. doi: 10.1371/journal.pone.0343234 (PMC12931771; doi:10.1371/journal.pone.0343234)
Supplement: S2 Table — (DOCX) [file pone.0343234.s002.docx]

Table S2 Baseline characteristics of study population according to the incidence of AKI

| **Variables** | **Total (n = 2820)** | **0 (n = 1737)** | **1 (n = 1083)** | **P** |
| --- | --- | --- | --- | --- |
| Age | 69.78 ± 13.84 | 69.64 ± 14.05 | 70.00 ± 13.49 | 0.494 |
| Gender, n(%) | 1678 (59.50) | 963 (55.44) | 715 (66.02) | <.001 |
| Race |  |  |  | 0.132 |
| WHITE | 1583 (56.13) | 983 (56.59) | 600 (55.40) |  |
| BLACK | 253 (8.97) | 141 (8.12) | 112 (10.34) |  |
| OTHER | 984 (34.89) | 613 (35.29) | 371 (34.26) |  |
| Length of hospitalization, days | 10.17 (6.15, 17.78) | 10.17 (6.23, 17.49) | 10.17 (5.59, 18.86) | 0.265 |
| Length of ICU, days | 3.81 (2.05, 7.00) | 3.62 (2.02, 6.61) | 5.06 (2.56, 9.70) | <.001 |
| Vital signs |  |  |  |  |
| Heart rate, bpm | 83.00 (72.00, 98.25) | 82.00 (71.00, 97.00) | 88.00 (74.00, 103.00) | <.001 |
| SBP, mmHg | 130.00 (112.00, 149.00) | 131.00 (113.00, 150.00) | 123.00 (107.25, 142.75) | <.001 |
| DBP, mmHg | 73.00 (62.00, 86.00) | 74.00 (63.00, 87.00) | 71.00 (59.00, 83.00) | <.001 |
| Respiratory rate, breaths /minute | 19.00 (16.00, 23.00) | 19.00 (16.00, 22.00) | 20.00 (16.00, 24.00) | <.001 |
| Spo2, % | 98.00 (95.00, 100.00) | 98.00 (95.00, 100.00) | 98.00 (95.00, 100.00) | 0.392 |
| Lab |  |  |  |  |
| Hemoglobin A1c | 5.90 (5.40, 6.80) | 5.90 (5.40, 6.80) | 6.00 (5.50, 6.90) | 0.005 |
| Glucose, mg/dL | 135.00 (108.00, 181.00) | 132.00 (106.00, 177.00) | 152.00 (120.00, 210.00) | <.001 |
| Hemoglobin, g/dL | 11.80 (10.00, 13.40) | 12.00 (10.30, 13.45) | 11.10 (9.30, 12.80) | <.001 |
| Platelet Count, K/uL | 207.00 (159.75, 265.00) | 208.00 (162.00, 263.00) | 203.00 (147.00, 272.00) | 0.282 |
| White Blood Cells, K/uL | 10.80 (8.10, 14.30) | 10.50 (8.00, 13.70) | 12.40 (8.90, 16.92) | <.001 |
| Potassium, mEq/L | 4.10 (3.80, 4.50) | 4.10 (3.77, 4.50) | 4.20 (3.80, 4.80) | <.001 |
| Sodium, mEq/L | 139.00 (136.00, 141.00) | 139.00 (136.00, 141.00) | 138.00 (135.00, 141.00) | 0.045 |
| Creatinine, mg/dl | 1.00 (0.80, 1.40) | 1.00 (0.80, 1.40) | 1.20 (0.90, 2.00) | <.001 |
| Urea Nitrogen, (mg/dL) | 19.00 (14.00, 30.00) | 18.00 (13.00, 28.00) | 26.00 (18.00, 45.00) | <.001 |
| GV (%) | 20.21 (14.16, 30.64) | 19.15 (13.61, 29.29) | 24.88 (17.13, 34.84) | <.001 |
| SHR | 1.07 (0.89, 1.33) | 1.05 (0.88, 1.30) | 1.19 (0.93, 1.50) | <.001 |
| HGI | 0.04 ± 1.55 | -0.00 ± 1.39 | 0.10 ± 1.74 | 0.037 |
| Scoring syatem |  |  |  |  |
| SOFA | 4.00 (2.00, 6.00) | 3.00 (2.00, 5.00) | 5.00 (3.00, 8.00) | <.001 |
| SAPSII | 35.00 (27.00, 43.00) | 33.00 (26.00, 41.00) | 41.00 (34.00, 50.00) | <.001 |
| Charlson | 6.00 (5.00, 8.00) | 6.00 (4.00, 8.00) | 7.00 (6.00, 9.00) | <.001 |
| Comorbidities, n(%) |  |  |  |  |
| Hypertension | 1250 (44.33) | 924 (53.20) | 326 (30.10) | <.001 |
| Diabetes | 1108 (39.29) | 603 (34.72) | 505 (46.63) | <.001 |
| Hyperlipidemia | 1466 (51.99) | 922 (53.08) | 544 (50.23) | 0.141 |
| COPD | 405 (14.36) | 215 (12.38) | 190 (17.54) | <.001 |
| Pneumonia | 827 (29.33) | 412 (23.72) | 415 (38.32) | <.001 |
| CKD | 657 (23.30) | 211 (12.15) | 446 (41.18) | <.001 |
| Cancer | 348 (12.34) | 204 (11.74) | 144 (13.30) | 0.223 |
| AKI Stage |  |  |  | <.001 |
| Stage 1 | 405 (20.83) | 219 (21.18) | 186 (20.44) |  |
| Stage 2 | 949 (48.82) | 583 (56.38) | 366 (40.22) |  |
| Stage 3 | 590 (30.35) | 232 (22.44) | 358 (39.34) |  |
| CRRT | 135 (4.79) | 23(1.32) | 112 (10.34) | <0.001 |
| Vasoactive agent | 1338(47.45) | 653(37.59) | 685(63.25) | <0.001 |
| Death rate | 505 (17.91) | 202 (11.63) | 303 (27.98) | <.001 |
