# Supplementary material for: Comparison of different glycemic control indicators on incidence of acute kidney injury and long-term mortality in critically ill patients with atherosclerotic cardiovascular disease: A retrospective cohort study
Source: PLoS One. 2026 Feb 24;21(2):e0343234. doi: 10.1371/journal.pone.0343234 (PMC12931771; doi:10.1371/journal.pone.0343234)
Supplement: S3 Table — (DOCX) [file pone.0343234.s003.docx]

Table S3 Association between SHR with AKI and long-term mortality in different subgroups.

| subgroups | | OR;95%CI | P value | P for interaction |  | HR;95%CI | P value | P for interaction |
| --- | --- | --- | --- | --- | --- | --- | --- | --- |
| Age |  |  |  | 0.56 |  |  |  | 0.11 |
| ≧60 | Q2 | ref |  |  | Q2 | Ref |  |  |
|  | Q1 | 0.86(0.66,1.13) | 0.28 |  | Q1 | 0.74(0.56,0.98) | 0.03 |  |
|  | Q3 | 0.97(0.74,1.26) | 0.80 |  | Q3 | 0.86(0.66,1.13) | 0.28 |  |
|  | Q4 | 1.61(1.24,2.10) | <0.001 |  | Q4 | 0.96(0.75,1.25) | 0.78 |  |
| <60 | Q2 | ref |  |  | Q2 | Ref |  |  |
|  | Q1 | 0.56(0.34,0.93) | 0.03 |  | Q1 | 1.16(0.63,2.14) | 0.63 |  |
|  | Q3 | 0.59(0.33,1.03) | 0.06 |  | Q3 | 1.29(0.67,2.48) | 0.45 |  |
|  | Q4 | 1.23(0.75,2.03) | 0.41 |  | Q4 | 1.04(0.54,2.00) | 0.91 |  |
| Sex |  |  |  | 0.72 |  |  |  | 0.45 |
| Male | Q2 | ref |  |  | Q2 | Ref |  |  |
|  | Q1 | 0.74(0.55,1.00) | 0.05 |  | Q1 | 0.82(0.59,1.14) | 0.24 |  |
|  | Q3 | 0.95(0.70,1.29) | 0.74 |  | Q3 | 0.88(0.63,1.22) | 0.44 |  |
|  | Q4 | 1.42(1.06,1.91) | 0.02 |  | Q4 | 1.08(0.80,1.46) | 0.62 |  |
| Female | Q2 | ref |  |  | Q2 | Ref |  |  |
|  | Q1 | 0.86(0.58,1.27) | 0.43 |  | Q1 | 0.76(0.51,1.13) | 0.17 |  |
|  | Q3 | 0.81(0.55,1.20) | 0.29 |  | Q3 | 0.98(0.67,1.43) | 0.92 |  |
|  | Q4 | 1.65(1.13,2.42) | 0.01 |  | Q4 | 0.85(0.58,1.26) | 0.42 |  |
| Diabetes |  |  |  | 0.63 |  |  |  | 0.56 |
| Yes | Q2 | ref |  |  | Q2 | Ref |  |  |
|  | Q1 | 0.89(0.54,1.48) | 0.66 |  | Q1 | 0.73(0.44,1.19) | 0.20 |  |
|  | Q3 | 0.8(0.51,1.26) | 0.34 |  | Q3 | 0.94(0.62,1.41) | 0.75 |  |
|  | Q4 | 1.11(0.74,1.65) | 0.61 |  | Q4 | 0.76(0.53,1.09) | 0.13 |  |
| No | Q2 | ref |  |  | Q2 | Ref |  |  |
|  | Q1 | 0.75(0.57,0.99) | 0.04 |  | Q1 | 0.83(0.61,1.11) | 0.21 |  |
|  | Q3 | 0.86(0.65,1.15) | 0.32 |  | Q3 | 0.89(0.65,1.22) | 0.47 |  |
|  | Q4 | 1.96(1.30,2.96) | 0.001 |  | Q4 | 1.35(0.90,2.01) | 0.15 |  |
| Hypertension | |  |  | 0.22 |  |  |  | 0.71 |
| Yes | Q2 | ref |  |  | Q2 | Ref |  |  |
|  | Q1 | 0.66(0.44,0.98) | 0.04 |  | Q1 | 1(0.66,1.54) | 0.98 |  |
|  | Q3 | 0.81(0.54,1.20) | 0.29 |  | Q3 | 1.22(0.80,1.86) | 0.35 |  |
|  | Q4 | 1.18(0.80,1.75) | 0.41 |  | Q4 | 1.29(0.85,1.95) | 0.23 |  |
| No | Q2 | ref |  |  | Q2 | Ref |  |  |
|  | Q1 | 0.91(0.68,1.23) | 0.55 |  | Q1 | 0.72(0.52,0.99) | 0.04 |  |
|  | Q3 | 1(0.74,1.35) | 0.98 |  | Q3 | 0.82(0.60,1.12) | 0.21 |  |
|  | Q4 | 1.87(1.40,2.51) | <0.0001 |  | Q4 | 0.88(0.66,1.18) | 0.38 |  |
| CKD |  |  |  | 0.47 |  |  |  | 0.21 |
| No | Q2 | ref |  |  | Q2 | Ref |  |  |
|  | Q1 | 0.69(0.52,0.92) | 0.01 |  | Q1 | 0.87(0.65,1.18) | 0.37 |  |
|  | Q3 | 0.85(0.63,1.13) | 0.26 |  | Q3 | 0.96(0.71,1.30) | 0.81 |  |
|  | Q4 | 1.43(1.07,1.90) | 0.01 |  | Q4 | 1.08(0.80,1.44) | 0.62 |  |
| Yes | Q2 | ref |  |  | Q2 | Ref |  |  |
|  | Q1 | 1.51(0.90,2.53) | 0.12 |  | Q1 | 0.65(0.40,1.06) | 0.08 |  |
|  | Q3 | 0.95(0.60,1.50) | 0.82 |  | Q3 | 0.85(0.55,1.30) | 0.45 |  |
|  | Q4 | 1.53(0.97,2.40) | 0.07 |  | Q4 | 0.76(0.51,1.15) | 0.19 |  |
