# Supplementary material for: Comparison of different glycemic control indicators on incidence of acute kidney injury and long-term mortality in critically ill patients with atherosclerotic cardiovascular disease: A retrospective cohort study
Source: PLoS One. 2026 Feb 24;21(2):e0343234. doi: 10.1371/journal.pone.0343234 (PMC12931771; doi:10.1371/journal.pone.0343234)
Supplement: S5 Table — (DOCX) [file pone.0343234.s005.docx]

Table S5 Association between GV with AKI and long-term mortality in different subgroups.

| subgroups |  | OR;95%CI | P value | p for interaction | | HR;95%CI | P value | p for interaction |
| --- | --- | --- | --- | --- | --- | --- | --- | --- |
| Age |  |  |  | 0.12 |  |  |  | 0.11 |
| ≧60 | Q1 | ref |  |  | Q1 | ref |  |  |
|  | Q2 | 1.83(1.36,2.47) | <0.0001 |  | Q2 | 1.32(0.95,1.83) | 0.10 |  |
|  | Q3 | 2.55(1.91,3.42) | <0.0001 |  | Q3 | 1.53(1.11,2.11) | 0.01 |  |
|  | Q4 | 4.41(3.28,5.93) | <0.0001 |  | Q4 | 1.74(1.27,2.39) | <0.001 |  |
| <60 | Q1 | ref |  |  | Q1 | ref |  |  |
|  | Q2 | 1.88(1.05,3.35) | 0.03 |  | Q2 | 1.81(0.75,4.33) | 0.18 |  |
|  | Q3 | 3.01(1.71,5.30) | <0.001 |  | Q3 | 3.27(1.47,7.30) | 0.004 |  |
|  | Q4 | 4.5(2.56,7.91) | <0.0001 |  | Q4 | 2.59(1.13,5.91) | 0.02 |  |
| Sex |  |  |  | 0.22 |  |  |  | 0.29 |
| Male | Q1 | ref |  |  | Q1 | ref |  |  |
|  | Q2 | 1.64(1.19,2.26) | 0.003 |  | Q2 | 1.31(0.88,1.95) | 0.18 |  |
|  | Q3 | 2.54(1.85,3.49) | <0.0001 |  | Q3 | 1.65(1.13,2.42) | 0.01 |  |
|  | Q4 | 4.12(2.98,5.68) | <0.0001 |  | Q4 | 1.88(1.29,2.73) | 0.001 |  |
| Female | Q1 | ref |  |  | Q1 | ref |  |  |
|  | Q2 | 2.62(1.60,4.28) | <0.001 |  | Q2 | 1.56(0.95,2.55) | 0.08 |  |
|  | Q3 | 3.4(2.10,5.51) | <0.0001 |  | Q3 | 1.99(1.24,3.19) | 0.004 |  |
|  | Q4 | 6.12(3.79,9.89) | <0.0001 |  | Q4 | 1.91(1.18,3.10) | 0.01 |  |
| Diabetes |  |  |  | 0.45 |  |  |  | 0.68 |
| Yes | Q1 | ref |  |  | Q1 | ref |  |  |
|  | Q2 | 1.33(0.79,2.24) | 0.28 |  | Q2 | 1.1(0.57,2.12) | 0.77 |  |
|  | Q3 | 1.85(1.17,2.94) | 0.01 |  | Q3 | 1.96(1.13,3.43) | 0.02 |  |
|  | Q4 | 3.07(1.95,4.83) | <0.0001 |  | Q4 | 1.49(0.85,2.61) | 0.16 |  |
| No | Q1 | ref |  |  | Q1 | ref |  |  |
|  | Q2 | 2.07(1.52,2.81) | <0.0001 |  | Q2 | 1.51(1.06,2.14) | 0.02 |  |
|  | Q3 | 2.98(2.15,4.13) | <0.0001 |  | Q3 | 1.47(1.01,2.14) | 0.04 |  |
|  | Q4 | 4.98(3.50,7.09) | <0.0001 |  | Q4 | 2.36(1.63,3.41) | <0.0001 |  |
| Hypertension | |  |  | 0.34 |  |  |  | 0.63 |
| Yes | Q1 | ref |  |  | Q1 | ref |  |  |
|  | Q2 | 1.09(0.71,1.67) | 0.70 |  | Q2 | 1.42(0.89,2.29) | 0.14 |  |
|  | Q3 | 2.17(1.44,3.27) | <0.001 |  | Q3 | 1.93(1.22,3.04) | 0.005 |  |
|  | Q4 | 3.45(2.28,5.23) | <0.0001 |  | Q4 | 2.12(1.33,3.36) | 0.002 |  |
| No | Q1 | ref |  |  | Q1 | ref |  |  |
|  | Q2 | 2.6(1.84,3.68) | <0.0001 |  | Q2 | 1.35(0.90,2.03) | 0.15 |  |
|  | Q3 | 2.95(2.10,4.13) | <0.0001 |  | Q3 | 1.63(1.11,2.41) | 0.01 |  |
|  | Q4 | 5.05(3.59,7.10) | <0.0001 |  | Q4 | 1.73(1.18,2.53) | 0.01 |  |
| CKD |  |  |  | 0.77 |  |  |  | 0.89 |
| No | Q1 | ref |  |  | Q1 | ref |  |  |
|  | Q2 | 1.85(1.32,2.59) | <0.001 |  | Q2 | 1.35(0.95,1.92) | 0.10 |  |
|  | Q3 | 3.23(2.33,4.48) | <0.0001 |  | Q3 | 1.61(1.14,2.27) | 0.01 |  |
|  | Q4 | 4.75(3.41,6.63) | <0.0001 |  | Q4 | 2.04(1.45,2.87) | <0.0001 |  |
| Yes | Q1 | ref |  |  | Q1 | ref |  |  |
|  | Q2 | 2.39(1.39,4.09) | 0.002 |  | Q2 | 1.61(0.84,3.05) | 0.15 |  |
|  | Q3 | 1.58(0.96,2.60) | 0.07 |  | Q3 | 2.14(1.18,3.89) | 0.01 |  |
|  | Q4 | 3.05(1.85,5.01) | <0.0001 |  | Q4 | 1.54(0.85,2.80) | 0.16 |  |
