# Supplementary material for: Comparison of different glycemic control indicators on incidence of acute kidney injury and long-term mortality in critically ill patients with atherosclerotic cardiovascular disease: A retrospective cohort study
Source: PLoS One. 2026 Feb 24;21(2):e0343234. doi: 10.1371/journal.pone.0343234 (PMC12931771; doi:10.1371/journal.pone.0343234)
Supplement: S6 Table — (DOCX) [file pone.0343234.s006.docx]

Table S6 Discrimination of each predictive model for outcomes

| Models | AUC (95% CI) | Cut-off | Sensitivity | Specificity | P-Value |
| --- | --- | --- | --- | --- | --- |
| AKI |  |  |  |  |  |
| SHR | 0.58 (0.56,0.60) | 1.41 | 0.29 | 0.86 | <0.001 |
| HGI | 0.60(0.58,0.62) | 0.04 | 0.39 | 0.41 | <0.001 |
| GV | 0.69(0.66,0.71) | 18.66 | 0.76 | 0.53 | <0.001 |
| Long-term mortality |  |  |  |  |  |
| SHR | 0.59(0.56,0.61) | 1.23 | 0.47 | 0.69 | <0.001 |
| HGI | 0.61(0.57,0.62) | 0.09 | 0.35 | 0.68 | <0.001 |
| GV | 0.62(0.58,0.63) | 21.53 | 0.65 | 0.54 | <0.001 |
