# Supplementary figures and images for: Comparison of different glycemic control indicators on incidence of acute kidney injury and long-term mortality in critically ill patients with atherosclerotic cardiovascular disease: A retrospective cohort study
Source: PLoS One. 2026 Feb 24;21(2):e0343234. doi: 10.1371/journal.pone.0343234 (PMC12931771; doi:10.1371/journal.pone.0343234)

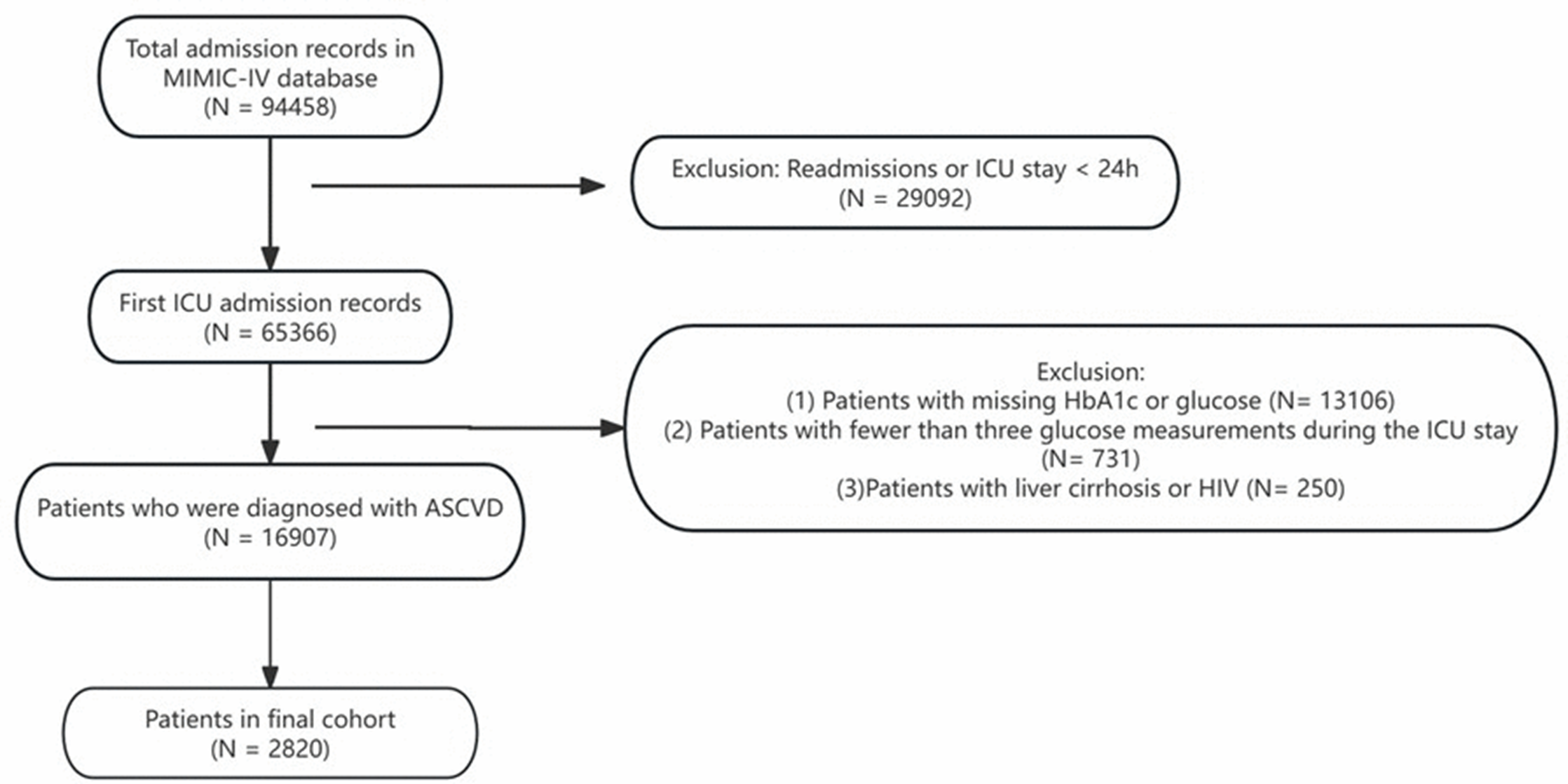

Supplement: S1 Fig — (TIF) [file pone.0343234.s007.tif]
